# Assessment of Heart Disease using Fuzzy Classification Techniques

**DOI:** 10.1100/tsw.2001.64

**Published:** 2001-08-17

**Authors:** Horia F. Pop, Tudor L. Pop, Costel Sarbu

**Affiliations:** Babes-Bolyai University, Faculty of Mathematics and Computer Science, RO-3400 Cluj-Napoca, Romania

**Keywords:** fuzzy clustering, characteristics clustering, fuzzy cross-clustering, characteristics selection, cardiac disease, ischemical cardiopathy, valvular heart disease, arterial hypertension

## Abstract

In this paper we discuss the classification results of cardiac patients of ischemical cardiopathy, valvular heart disease, and arterial hypertension, based on 19 characteristics (descriptors) including ECHO data, effort testings, and age and weight. In this order we have used different fuzzy clustering algorithms, namely hierarchical fuzzy clustering, hierarchical and horizontal fuzzy characteristics clustering, and a new clustering technique, fuzzy hierarchical cross-classification. The characteristics clustering techniques produce fuzzy partitions of the characteristics involved and, thus, are useful tools for studying the similarities between different characteristics and for essential characteristics selection. The cross-classification algorithm produces not only a fuzzy partition of the cardiac patients analyzed, but also a fuzzy partition of their considered characteristics. In this way it is possible to identify which characteristics are responsible for the similarities or dissimilarities observed between different groups of patients.

### INTRODUCTION

The mathematics of fuzzy set theory was originated by L.A. Zadeh in 1965[15]. It deals with the uncertainty and fuzziness arising from interrelated humanistic types of phenomena such as subjectivity, thinking, reasoning, cognition and perception. This type of uncertainty is characterized by structures that lack sharp (well-defined) boundaries. This approach provides a way to translate a linguistic model of the human thinking process into a mathematical framework for developing the computer algorithms for computerized decision-making processes. The theory has grown very quickly[1,2,3,5].

There are two opposite approaches to hierarchical clustering, namely, *agglomerative* and *divisive* procedures. An agglomerative hierarchical classification places each object in its own cluster and gradually merges the clusters into larger and larger clusters until all objects are in a single cluster. The divisive hierarchical clustering reverses the process by starting with all the objects in a single cluster and subdividing it into smaller ones until, finally, each object is in a cluster of its own. The number of clusters to be generated may be either specified in advance or optimized by the algorithm itself according to certain criteria.

Among other interesting applications, the fuzzy clustering theory developed in References [3,4,6,8] has been used for the selection and the optimal combination of solvents[7,13], for the classification of Roman pottery[9], for the cross-classification of Greek muds[6], for the development of a fuzzy system of chemical elements[12,14], for producing a performant fuzzy regression algorithm[10], and for the cross-classification of thin layer chromatography data[11].

In this paper we analyze the possibility of identifying the correct diagnosis concerning the cardiac patients using different fuzzy clustering algorithms.

### THEORETICAL CONSIDERATIONS

#### Fuzzy Substructure of a Fuzzy Set

In this section we will recall the so-called generalized fuzzy *n*-means algorithm[3,4,6]. This algorithm is a generalization of the well-known fuzzy *n*-means algorithm[1,4]. Let us consider a set of objects $X = \{ x^1, ..., x^p \} \subset \mathbf{R}^s$ and let $C$ be a fuzzy set on $X$. We are searching for the fuzzy partition corresponding to the cluster substructure of the fuzzy set $C$. Let us suppose that this fuzzy partition is $\{A_1, ..., A_n\}$. We admit that each fuzzy class $A_i$ may be represented by a prototype $L^i$ from the representation space, $\mathbf{R}^s$. If $L^i$ is from $X$, it is natural to suppose that $L^i$ has the greatest membership degree to $A_i$, that is:

$$A_i(L^i) = \max_{x \in X} A_i(x) \tag{1}$$

Otherwise, if $L^i$ is not from $X$, we cannot speak about its membership degree to the fuzzy set $A_i$, since the universe of the fuzzy set $A_i$ is $X$.

Let us denote by $d$ a distance in the space $\mathbf{R}^s$. For example, we may consider the distance induced by the norm of the space. The dissimilarity $D_i(x^j, L^i)$ between a point $x^j$ and the prototype $L^i$ is defined as the square local distance in the class $A_i$, $d_i(x^j, L^i)$, and is interpreted as a measure of the inadequacy of the representation of the point $x^j$ by the prototype $L^i$.

If $L^i$ is not a point from the data set $X$, then

$$D_i(x^j, L^i) = (A_i(x^j))^2 d^2(x^j, L^i). \tag{2}$$

If, on the contrary, $L^i$ is a point from the data set $X$, then we have from (1) that $A_i(L^i) \geq A_i(x^j)$ for any $x^j$ in $X$, and the relation (2) is still valid.

The inadequacy between the fuzzy partition $P$ and its representation, $L = \{L^1, ..., L^n\}$ is given by the following function:

$$J(P,L)=\sum_{i=1}^{n}\sum_{j=1}^{p}(A_i(x^j))^2 d^2(x^j,L^i). \tag{3}$$

$J(P, L)$ may also be interpreted as the representation error of $P$ by $L$.

It is easy to observe that $J$ is a criteria function of the type of square errors sum. The classification problem becomes the determination of the fuzzy partition $P$ and its representation $L$ for which the inadequacy $J(P, L)$ is minimal. We note that, intuitively, to minimize $J$ means to give small membership degrees to $A_i$ for those points in $X$ for which the dissimilarity to the prototype $L^i$ is large, and vice-versa. Another useful remark is that the fuzzy sets $A_i$, $i = 1,...,n$ are components of the fuzzy partition $P$ of the fuzzy set $C$, and thus the obvious 'solution', $A_i = 0$ for all $i$ is not acceptable since it does not form a fuzzy partition of $C$.

If we admit that $d$ is a distance induced by the norm, we may write

$$J(P,L)=\sum_{i=1}^{n}\sum_{j=1}^{p}(A_i(x^j))^2\left\|x^j-L^i\right\|^2. \tag{4}$$

If the norm is induced by the inner product, we have

$$\| x^j - L^i \|^2 = (x^j - L^i)^T M (x^j - L^i),$$

where $M$ is a symmetrical and positively defined matrix. The transposing operation was denoted by $^T$. The criteria function becomes:

$$J(P,L)=\sum_{i=1}^{n}\sum_{j=1}^{p}(A_i(x^j))^2\left(x^j-L^i\right)^T M\left(x^j-L^i\right). \tag{5}$$

Because an algorithm to obtain an exact solution of the problem (5) is not known, we will use an approximate method in order to determine a local solution. The minimum problem will be solved using an iterative (relaxation) method, where $J$ is successively minimized with respect to $P$ and $L$.

Supposing that $L$ is given, the minimum of the function $J(\bullet,L)$ is obtained[3, 4] for:

$$A_i(x^j)=\frac{C(x^j)}{\sum_{k=1}^{n}\frac{d^2(x^j,L^i)}{d^2(x^j,L^k)}}, i=1,...,n, \tag{6}$$

for all $j$ for which for every $k$, $d(x^j, L^k) \neq 0$. If, on the contrary, there exists a $j$ so that for some values of $k$, $d(x^j, L^k) = 0$, then the membership degrees $A_i(x^j)$ fulfill the condition $A_i(x^j) = 0$ for all $i$ so that $d(x^j, L^i) \neq 0$.

For a given $P$, the minimum of the function $J(P,\cdot)$ is obtained for:

$$L^i=\frac{\sum_{j=1}^{p}A_i(x^j)^2 x^j}{\sum_{j=1}^{p}A_i(x^j)^2}, i=1,...,n \tag{7}$$

We observe[3,4] that $L^i$ is the weighting center of the class $A_i$.

The iterative procedure for obtaining the cluster substructure of the fuzzy class $C$ is called generalized fuzzy $n$-means (GFNM)[3]. Essentially, the GFNM algorithm works with Picard iterations using the relation (6) and (7). The iterative process begins with an arbitrary initialization of the partition $P$. The process ends when two successive partitions are close enough. To measure the distance between two partitions, we will associate to each partition $P$ a matrix $Q$ with the dimensions $n \times p$. $Q$ is named the representation matrix of the fuzzy partition $P$ and is defined as:

$$Q_{ij} = A_i(x^j),\ i = 1, 2, ..., n;\ j = 1, 2, ..., p. \qquad (8)$$

Considering that $Q_1$ and $Q_2$ are the representation matrices of the partitions $P_1$ and $P_2$, we may define

$$d(P_1, P_2) = ||Q_1 - Q_2|| \qquad (9)$$

where $\|Q\| = \max_{i,j} \left| A_i(x^j) \right|$.

The process ends at the $r$-th iteration if

$$d(P_r, P_{r+1}) < \varepsilon \qquad (10)$$

where $\varepsilon$ is an admissible error (usually, $10^{-5}$).

For $C = X$ this procedure is the well-known algorithm fuzzy $n$-means (FNM)[1].

### Fuzzy Divisive Hierarchical Clustering

Let us consider a fuzzy set $C$ on $X$ and a fuzzy binary partition $P = \{A_1, A_2\}$ of $C$, and the following function, called polarization degree:

$$R(P) = \frac{\sum_{x \in X} \left( A_{1,1/2}(x) + A_{2,1/2}(x) \right)}{\sum_{x \in X} C(x)}, \qquad (11)$$

where $A_{i,1/2}$ is the 1/2-cut of $A_i$ (i.e., $A_{i,1/2}(x)$ is equal to $A_i(x)$ if $A_i(x)$ is larger than $C(x)/2$ and is equal to zero otherwise). (For a complete study of the polarization degree and its properties, please see Reference [3].) We will say here only that $R(P)$ is as larger as the partition $P$ is more polarized, and $R(P)$ is as small as the partition $P$ is fuzzy. We say that the binary partition $P$ describes 'real' clusters if the polarization degree $R(P)$ is larger than a certain threshold $t \in (0,1)$ chosen *a priori,* and if for every class $C_i$ there exists at least one point $x$ in $X$ so that $A_i(x) > C(x)/2$.

Using the FNM algorithm we may determine a binary fuzzy partition $P = \{A_1, A_2\}$ of the data set $X$. If the partition $P$ does not describe 'real' clusters, the data set $X$ does not have a substructure. If this partition describes 'real' clusters, we denote $P^1 = \{A_1, A_2\}$. Using the GFNM algorithm for two subclasses ($n = 2$) we may determine a binary fuzzy partition for each $A_i$ of $P^1$. If this partition of $A_i$ describes real clusters, these clusters will be attached to a new fuzzy partition, $P^2$. Otherwise, $A_i$ will remain undivided. The class $A_i$ will be marked and will be allocated to the partition $P^2$. The unmarked classes members of $P^2$ will follow the same procedure. The divisive procedure will stop when all the classes of the current partition $P^l$ are marked, i.e., there are no more 'real' clusters.

The procedure described here is called the Fuzzy Divisive Hierarchical Clustering (FDHC) algorithm[3,9]. This procedure may be used to determine the optimal cluster substructure of the data set. The method is especially useful when the number of classes is unknown. We emphasize here that the ability of chose the value of the polarization threshold to be used allows us to stop the hierarchical analysis at that degree of refinement considered relevant for the application. If we decide to choose a high threshold we will obtain the fuzzy partition corresponding to the macroscopic structure of the data set, while by choosing a smaller threshold, we will have a more detailed image of the fuzzy substructure of the data. Moreover, we are not interested only in the final fuzzy partition; we are interested in the relationships between different fuzzy sets. These relationships may be observed very well from the binary classification tree[6,7,9,11,12,14].

### Interpretation of the Final Fuzzy Partition

The fuzzy hierarchy obtained is richer in information (see Reference [5]) than a hierarchy based on classical sets, but sometimes is useful to have a classical partition also. For a complete discussion on the problem of passing from fuzzy partitions to classical partitions, see Reference [5]. We will only show the method used here for obtaining a classical partition.

Defuzzification of the final fuzzy partition will be realized using the maximum membership rule or a hierarchical assignment rule. This latter rule means that the classical sets corresponding to the fuzzy classes will be built in the same time with the respective fuzzy classes, based on the following rules (here, and in all that follows, $\tilde{C}$ denotes the classical set obtained by defuzzification from the fuzzy set $C$): 1) initially, since $X$ is a classical set, $\tilde{X} = X$; 2) when we build the fuzzy partition $\{C_1, C_2\}$ of the fuzzy set $C$, we will say that:

$$x \in \tilde{C}_1 \Leftrightarrow \left(x \in \tilde{C} \text{ and } C_1(\mathrm{x}) \geq \mathrm{C}_2(\mathrm{x})\right)$$

and

$$x \in \tilde{C}_2 \Leftrightarrow \left(x \in \tilde{C} \text{ and } C_1(\mathrm{x}) < \mathrm{C}_2(\mathrm{x})\right)$$

**Remark.** It is obvious that $\{\tilde{C}_1, \tilde{C}_2\}$ is a hard partition of the classical set $\tilde{C}$.

Finally, when obtaining the fuzzy hierarchy of the set $X$, we will also obtain the so-called classical hierarchy associated to that fuzzy hierarchy.

### Associative Simultaneous Fuzzy *n*-Means Algorithm

Let $X = \{x^1, ..., x^p\} \subset \mathbf{R}^s$ be the set of objects to be classified. A characteristic may be specified by its values corresponded to the $p$ objects. Thus, we may say that $Y = \{y^1, ..., y^s\} \subset \mathbf{R}^p$ is the set of characteristics. $y_j^k$ is the value of the characteristic $k$ with respect to the object $j$, so we may write $y_j^k = x_k^j$.

Let $P$ be a fuzzy partition of the fuzzy set $C$ of objects and $Q$ a fuzzy partition of the fuzzy set $D$ of characteristics. The problem of the cross-classification (or simultaneous classification) is to determine the pair ($P$, $Q$) which optimizes a certain criterion function.

By starting with an initial partition $P^0$ of $C$ and an initial partition $Q^0$ of $D$, we will obtain a new partition $P^1$. The pair ($P^1$, $Q^0$) allows us to determine a new partition $Q^1$ of the characteristics. The algorithm consists in producing a sequence $(P^k, Q^k)$ of pairs of partitions, starting from the initial pair $(P^0, Q^0)$, in the following steps:

**(i)** $(P^k, Q^k) \to (P^{k+1}, Q^k)$;
**(ii)** $(P^{k+1}, Q^k) \to (P^{k+1}, Q^{k+1})$.

The rationale of the hierarchical cross-classification method[4,6] essentially supposes the splitting of the sets $X$ and $Y$ in two subclasses. The classes obtained are alternatively divided in two subclasses, and so on. The two hierarchies will be represented by the same tree, having in each node a pair $(C, D)$, where $C$ is an objects fuzzy set and $D$ is a characteristics fuzzy set.

As a first step we wish to determine simultaneously the fuzzy partitions (as a particular case, the binary fuzzy partitions) of the classes $C$ and $D$, so that the two partitions should be highly correlated. With the generalized fuzzy $n$-means algorithm, we will determine a fuzzy partition $P = \{A_1, ..., A_n\}$ of the class $C$, using the original characteristics.

In order to classify the characteristics, we will compute their values for the classes $A_i$, $i = 1, ..., n$. The value $\bar{y}_i^k$ of the characteristic $k$ with respect to the class $A_i$ is defined as:

$$\bar{y}_i^k = \sum_{j=1}^{p} A_i(x^j) x_k^j, i = 1,...,n; k = 1,...,s. \tag{12}$$

Thus, from the original $s$ $p$-dimensional characteristics we computed $s$ new $n$-dimensional characteristics which are conditioned by the classes $A_i$, $i = 1, ..., n$. We may admit that these new characteristics do not describe objects, but they characterize the classes $A_i$.

Let us consider now the set $\bar{Y} = \{\bar{y}^1,...,\bar{y}^s\}$ of the modified characteristics. We define the fuzzy set $\bar{D}$ on $\bar{Y}$ given by

$$\bar{D}(\bar{y}^k) = D(y^k), k = 1,...,s.$$

The way the set $\bar{Y}$ has been obtained lets us conclude that if we will obtain an optimal partition of the fuzzy set $D$, this partition will be highly correlated to the optimal partition of the class $C$. With the generalized fuzzy $n$-means algorithm we will determine a fuzzy partition $Q = \{B_1, ..., B_n\}$ of the class $D$, by using the characteristics given by the relation (12). We may now characterize the objects in $X$ with respect to the classes of properties $B_i$, $i = 1, ..., n$. The value $\bar{x}_i^j$ of the object $j$ with respect to the class $B_i$ is defined as:

$$\bar{x}_i^j = \sum_{k=1}^{d} B_i(\bar{y}^k) x_k^j, i = 1,...,n; j = 1,..., p. \tag{13}$$

Thus, from the original $p$ $s$-dimensional objects we have computed $p$ new $n$-dimensional objects, which correspond to the classes of characteristics $B_i$, $i = 1, ..., n$.

Let us consider now the set $\bar{X} = \{\bar{x}^1,...,\bar{x}^p\}$ of the modified characteristics. We define the fuzzy set $\bar{C}$ on $\bar{X}$ given by

$$\bar{C}(\bar{x}^j) = C(x^j), j = 1,..., p.$$

With the generalized fuzzy $n$-means algorithm we will determine a fuzzy partition $P' = \{A'_1, ..., A'_n\}$, of the class $C$ by using the objects given by the relation (13). The process continues until two successive partitions of objects (or characteristics) are closed enough to each other.

Considering $P = \{A_1, ..., A_n\}$ is the fuzzy $n$-partition of $X$ and $Q = \{B_1, ..., B_n\}$ is the fuzzy $n$-partition of $Y$ produced after this step of our algorithm. Let us remark that we made no explicit association of a fuzzy set $A_i$ on $X$ with a fuzzy set $B_j$ on $Y$, i.e., what is the fuzzy set $B_j$ that best describes the essential characteristics corresponding to the fuzzy set $A_i$. It only supposes that $A_i$ is to be associated with $B_i$, and this is not always true.

Let us denote by $S_n$ the set of all permutations on $\{1, ..., n\}$. We wish to build that permutation $\sigma \in S_n$ which best associates the fuzzy set $A_i$ with the fuzzy set $B_{\sigma(i)}$, for every $i = 1, ..., n$. Our aim is to build some function $J : S_n \rightarrow \mathbf{R}$ so that the optimal permutation $\sigma$ is that which maximizes this function. Let us consider the matrix $Z \in \mathbf{R}^{n,n}$ given by

$$z_{kl} = \sum_{j=1}^{p} \sum_{i=1}^{d} A_k(x^j) B_l(y^i) x_i^j, k, l = 1, ..., n. \qquad (14)$$

Let us remark the similarity between the way we compute the matrix $Z$ in (14) and the way we computed the new objects and characteristics in relation (12) and (13).

The experience enables us to consider the function $J$ as given by

$$J = \prod_{i=1}^{n} z_{i,\sigma(i)}. \qquad (15)$$

Thus, supposing that the permutation $\sigma$ maximizes the function $J$ defined above, we will be able to associate the fuzzy set $A_i$ with the fuzzy set $B_{\sigma(i)}$, $i = 1, ..., n$. As we will see in the comparative study below, this association is more natural than the association of $A_i$ with $B_i$, $i = 1, ..., n$. Based on these considerations we are able to introduce the following algorithm, the associative simultaneous fuzzy $n$-means algorithm (ASF):

**S1.** Set $l = 0$. With the generalized fuzzy $n$-means algorithm we determine a fuzzy $n$-partition $P^{(l)}$ of the class $C$ by using the initial objects.

**S2.** With the generalized fuzzy $n$-means algorithm we determine a fuzzy $n$-partition $Q^{(l)}$ of the class $D$ by using the characteristics defined in (12).

**S3.** With the generalized fuzzy $n$-means algorithm we determine a fuzzy $n$-partition $P^{(l+1)}$ of the class $C$ by using the objects defined in (13).

**S4.** If the partitions $P^{(l)}$ and $P^{(l+1)}$ are close enough, that is, if $\|P^{(l+1)} - P^{(l)}\| < \varepsilon$, where $\varepsilon$ is a preset value, then go to **S5**, otherwise increase $l$ by 1 and go to **S2**.

**S5.** Compute the permutation $\sigma$ that maximizes the function $J$ given in relation (15).

**S6.** Re-label the fuzzy sets $B_i$, so that $B_{\sigma(i)}$ becomes $B_i$, $i = 1, ..., n$.

Let us remark now that, after steps **S5** and **S6**, we are able to associate the fuzzy set $A_i$ with the fuzzy set $B_i$, $i = 1, ..., n$.

Let us also remark that the computation required in step **S5** is not an obvious one. But, as we will see, our purpose is to use this algorithm for developing a hierarchical technique. Thus, we will use the ASF algorithm in the particular case $n = 2$. In this case, the computation required in step **S5** becomes trivial.

### Fuzzy Hierarchical Cross-Classification Algorithm

The method described below is the straightforward way of developing a hierarchical algorithm that should use at each node of the classification tree our ASF algorithm. We will first show the way to build the classification binary tree. The tree nodes are labeled with a pair ($C$, $D$), where $C$ is a fuzzy set from a fuzzy partition of objects and $D$ is a fuzzy set from a fuzzy partition of characteristics. The root node corresponds to the pair ($X$, $Y$). In the first step the two sub-nodes ($A_1$, $B_1$) and ($A_2$, $B_2$), respectively, will be computed by using the ASF algorithm. Of course, these two nodes will be effectively built only if the fuzzy partitions $\{A_1, A_2\}$ and $\{B_1, B_2\}$ describe real clusters. For each of the terminal nodes of the tree we try to determine partitions having the form $\{A_1, A_2\}$ and $\{B_1, B_2\}$, by using the ASF algorithm, modified as we have mentioned before. In this way the binary classification tree is extended with two new nodes, ($A_1$, $B_1$) and ($A_2$, $B_2$). The process continues until, for any terminal node, we are not able to determine a structure of real clusters, either for the set of objects or for the set of characteristics. The final fuzzy partitions will contain the fuzzy sets corresponding to the terminal nodes of the binary classification tree. This algorithm, termed the FHCCA algorithm, seems to be suitable for applications where the idea is to get most of the relationships between different classes of objects and different classes of characteristics. In Pop and Sârbu[11] we introduced two more variants of this algorithm, FHCCB and FHCCC.

### Characteristics Clustering

In this section we address the problem of characteristics clustering. This may be useful in many situations. For example, the dimensionality reduction may be considered a characteristic classification process. The characteristics in the same class (which are, consequently, very similar to each other) will realize a reduced discrimination among the objects. On the contrary, the more distant the classes that contain two different characteristics, the greater their discrimination power. If the classes of characteristics are homogenous and well separated, a class may be replaced by the most representative characteristic. This characteristic represents an average of the properties of the class. The more compact the class, the smaller the loss of information produced by this replacement. In this way we realize a dimensionality reduction. By choosing a unique characteristic from each class, the number of selected characteristics is equal to the number of clusters in the set $Y$. Alternatively, we may not only select some of the existing characteristics, but we may replace them by new characteristics, by considering that each class of characteristics is replaced by the prototype characteristic. The technique obtained by using the fuzzy divisive hierarchical clustering algorithm on the set of characteristics will be called fuzzy hierarchical characteristics clustering (FHiCC). Similarly, the technique obtained by using the fuzzy $n$-means algorithm on the set of characteristics will be called fuzzy horizontal characteristics clustering (FHoCC).

## CLASSIFICATION RESULTS AND DISCUSSIONS

### Analysis of Human Experts

The data set analyzed in this paper consists of 72 cardiac patients described by 19 variables (descriptors), as follows: age (1), weight (2), ECHO data including left ventricle (3) and (4), right ventricle (5), left atrium (6) and contractility (7) and (8), respectively and effort testings for level of effort (9), duration (10), cardiac frequency (11), maximum cardiac frequency (12), systolic arterial tension (13), diastolic arterial tension (14), $VTO_2$ (MET) (15), index of tension-time (16), relative index of tension-time (17), body aerobic deficit (18), and myocardium aerobic deficit (19). Tables 1 and 2 show the values of these 19 descriptors for 72 patients.

**TABLE 1**
**Original Values of the 19 Descriptors for 72 Cardiac Patients (Part I)**

| Patient | Characteristic (descriptor) | | | | | | | | | |
|---|---|---|---|---|---|---|---|---|---|---|
| | 1 | 2 | 3 | 4 | 5 | 6 | 7 | 8 | 9 | 10 |
| 1 | 49 | 58 | 50 | 35 | 14 | 35 | 67 | 31 | 100 | 150 |
| 2 | 43 | 65 | 55 | 40 | 18 | 42 | 61 | 27.2 | 50 | 167 |
| 3 | 42 | 73 | 60 | 45 | 19 | 45 | 58 | 22 | 75 | 115 |
| 4 | 53 | 87 | 53 | 40 | 19 | 30 | 63 | 24 | 100 | 100 |
| 5 | 33 | 91 | 58 | 40 | 18 | 35 | 68 | 31 | 75 | 125 |
| 6 | 65 | 89 | 55 | 40 | 14 | 30 | 61 | 27.2 | 50 | 107 |
| 7 | 39 | 67 | 52 | 35 | 21 | 35 | 68 | 32 | 125 | 150 |
| 8 | 67 | 85 | 50 | 38 | 15 | 28 | 56.1 | 24 | 100 | 136 |
| 9 | 48 | 75 | 62 | 40 | 15 | 47 | 71 | 34 | 100 | 150 |
| 10 | 36 | 70 | 59 | 45 | 15 | 32 | 57 | 23.7 | 75 | 125 |
| 11 | 49 | 59 | 50 | 30 | 14 | 28 | 79 | 41 | 50 | 115 |
| 12 | 41 | 67 | 50 | 35 | 21 | 30 | 67 | 31 | 75 | 162 |
| 13 | 57 | 65 | 65 | 50 | 20 | 50 | 54 | 23 | 100 | 115 |
| 14 | 28 | 70 | 56 | 40 | 19 | 30 | 55 | 23 | 125 | 162 |
| 15 | 56 | 67 | 48 | 30 | 21 | 33 | 73 | 36 | 75 | 136 |
| 16 | 32 | 76 | 62 | 48 | 18 | 38 | 53.5 | 22.5 | 100 | 125 |
| 17 | 41 | 73 | 58 | 43 | 20 | 41 | 58 | 25 | 75 | 125 |
| 18 | 27 | 55 | 50 | 30 | 14 | 31 | 79 | 41 | 100 | 167 |
| 19 | 46 | 67 | 47 | 26 | 15 | 41 | 83.07 | 44.6 | 100 | 152 |
| 20 | 44 | 71 | 60 | 45 | 20 | 48 | 25 | 58 | 125 | 136 |
| 21 | 33 | 61 | 49 | 30 | 20 | 41 | 78 | 40 | 100 | 167 |
| 22 | 44 | 86 | 60 | 45 | 20 | 40 | 58 | 25 | 150 | 167 |
| 23 | 39 | 66 | 55 | 40 | 20 | 52 | 61 | 27.2 | 50 | 100 |
| 24 | 40 | 73 | 49 | 35 | 16 | 33 | 66 | 30 | 100 | 136 |
| 25 | 40 | 60 | 57 | 40 | 14 | 40 | 63 | 29.8 | 125 | 167 |
| 26 | 60 | 88 | 62 | 50 | 22 | 50 | 44 | 17 | 125 | 150 |
| 27 | 48 | 77 | 48 | 31 | 29 | 50 | 73 | 35.4 | 75 | 88 |
| 28 | 56 | 56 | 60 | 40 | 20 | 38 | 70 | 33.3 | 50 | 125 |
| 29 | 50 | 74 | 80 | 65 | 30 | 60 | 46.3 | 18.75 | 50 | 100 |
| 30 | 50 | 76 | 49 | 30 | 18 | 30 | 74 | 37 | 100 | 100 |
| 31 | 49 | 87 | 58 | 45 | 18 | 42 | 55 | 20 | 150 | 157 |
| 32 | 59 | 82 | 55 | 35 | 18 | 50 | 70 | 36 | 75 | 157 |
| 33 | 43 | 68 | 53 | 40 | 21 | 48 | 57 | 24.5 | 75 | 83 |
| 34 | 56 | 55 | 55 | 40 | 19 | 50 | 61 | 27.2 | 75 | 100 |
| 35 | 42 | 68 | 62 | 45 | 20 | 35 | 59 | 26 | 100 | 94 |
| 36 | 33 | 68 | 52 | 30 | 20 | 42 | 80 | 42 | 75 | 150 |
| 37 | 45 | 76 | 50 | 35 | 18 | 39 | 67 | 31 | 50 | 136 |
| 38 | 44 | 86 | 54 | 40 | 19 | 43 | 61 | 27 | 100 | 167 |
| 39 | 48 | 75 | 68 | 52 | 30 | 54 | 42 | 21 | 50 | 136 |
| 40 | 57 | 63 | 60 | 45 | 20 | 42 | 58 | 25 | 50 | 100 |
| 41 | 31 | 60 | 94 | 80 | 25 | 65 | 38.3 | 15.6 | 50 | 147 |
| 42 | 67 | 60 | 74 | 62 | 30 | 50 | 28 | 13.8 | 25 | 150 |
| 43 | 36 | 80 | 74 | 65 | 14 | 40 | 25.3 | 12.1 | 100 | 150 |
| 44 | 50 | 78 | 86 | 76 | 20 | 64 | 29 | 11.6 | 50 | 115 |
| 45 | 58 | 60 | 87 | 76 | 20 | 50 | 30 | 12 | 25 | 80 |
| 46 | 69 | 100 | 80 | 65 | 30 | 48 | 46 | 18.75 | 50 | 94 |
| 47 | 53 | 88 | 65 | 47 | 34 | 45 | 62.1 | 27.6 | 75 | 160 |
| 48 | 55 | 100 | 90 | 76 | 40 | 70 | 39.7 | 15.5 | 25 | 118 |
| 49 | 54 | 90 | 47 | 30 | 20 | 30 | 73.9 | 36 | 100 | 150 |

| | | | | | | | | | | |
|---|---|---|---|---|---|---|---|---|---|---|
| 50 | 47 | 70 | 55 | 40 | 9 | 30 | 61 | 27.2 | 75 | 100 |
| 51 | 50 | 90 | 55 | 40 | 10 | 33 | 61 | 27.2 | 100 | 150 |
| 52 | 63 | 90 | 72 | 60 | 20 | 55 | 42 | 16.6 | 50 | 115 |
| 53 | 52 | 95 | 53 | 40 | 17 | 40 | 56 | 24 | 100 | 150 |
| 54 | 55 | 80 | 75 | 55 | 20 | 45 | 60 | 26.6 | 50 | 85 |
| 55 | 46 | 75 | 65 | 55 | 15 | 40 | 40 | 15.3 | 100 | 110 |
| 56 | 69 | 70 | 65 | 50 | 20 | 42 | 54 | 23 | 50 | 94 |
| 57 | 54 | 68 | 51 | 30 | 17 | 30 | 79.6 | 41 | 50 | 167 |
| 58 | 73 | 60 | 76 | 65 | 34 | 65 | 37 | 14.4 | 25 | 115 |
| 59 | 56 | 85 | 51 | 35 | 19 | 30 | 67.6 | 31.3 | 125 | 136 |
| 60 | 58 | 80 | 65 | 50 | 22 | 40 | 54 | 23 | 125 | 115 |
| 61 | 56 | 85 | 50 | 35 | 20 | 30 | 67 | 31 | 50 | 120 |
| 62 | 51 | 105 | 65 | 50 | 18 | 42 | 54 | 23 | 50 | 136 |
| 63 | 68 | 88 | 60 | 50 | 20 | 40 | 43 | 16 | 75 | 125 |
| 64 | 52 | 79 | 60 | 50 | 20 | 40 | 43 | 16 | 50 | 136 |
| 65 | 43 | 64 | 60 | 45 | 20 | 30 | 57.8 | 25 | 50 | 125 |
| 66 | 49 | 89 | 58 | 34 | 20 | 45 | 79.8 | 41.3 | 100 | 170 |
| 67 | 58 | 70 | 65 | 55 | 20 | 42 | 43 | 16 | 75 | 107 |
| 68 | 68 | 50 | 45 | 30 | 22 | 25 | 70.3 | 33.3 | 75 | 100 |
| 69 | 52 | 85 | 60 | 45 | 18 | 30 | 58 | 25 | 75 | 125 |
| 70 | 41 | 70 | 50 | 35 | 16 | 32 | 67 | 31 | 75 | 168 |
| 71 | 46 | 80 | 62 | 50 | 20 | 45 | 44 | 17 | 100 | 130 |
| 72 | 47 | 90 | 55 | 45 | 18 | 35 | 45.2 | 18.18 | 75 | 150 |

**TABLE 2**
**Original Values of the 19 Descriptors for 72 Cardiac Patients (Part II)**

| | Characteristic (descriptor) | | | | | | | | |
|---|---|---|---|---|---|---|---|---|---|
| **Patient** | **11** | **12** | **13** | **14** | **15** | **16** | **17** | **18** | **19** |
| 1 | 161 | 190 | 100 | 25 | 7.14 | 31500 | 28500 | 22.81 | 16.46 |
| 2 | 167 | 135 | 85 | 14 | 4 | 32100 | 22545 | 60.99 | 33.50 |
| 3 | 178 | 155 | 90 | 17 | 4.85 | 32200 | 17825 | 52.86 | 44.51 |
| 4 | 167 | 155 | 85 | 16 | 4.57 | 31100 | 15500 | 53.20 | 50.16 |
| 5 | 177 | 150 | 90 | 13.3 | 3.8 | 33100 | 18750 | 66.90 | 45.63 |
| 6 | 145 | 175 | 90 | 10 | 2.85 | 29900 | 18725 | 68.89 | 42.64 |
| 7 | 171 | 230 | 110 | 26 | 7.42 | 32500 | 34500 | 17.96 | 1.06 |
| 8 | 153 | 180 | 95 | 20 | 5.71 | 29700 | 24480 | 46.55 | 24.70 |
| 9 | 162 | 165 | 90 | 20 | 5.71 | 31600 | 24750 | 42.92 | 26.37 |
| 10 | 174 | 115 | 80 | 17 | 4.85 | 32800 | 14375 | 63.45 | 68.20 |
| 11 | 161 | 150 | 90 | 15 | 4.28 | 31500 | 17250 | 56.98 | 48.59 |
| 12 | 169 | 175 | 90 | 18 | 5.14 | 32300 | 28350 | 53.07 | 16.67 |
| 13 | 163 | 190 | 100 | 23 | 6.57 | 30700 | 21850 | 31.36 | 28.82 |
| 14 | 182 | 155 | 90 | 25.5 | 7.28 | 33600 | 25885 | 34.96 | 25.56 |
| 15 | 154 | 165 | 90 | 17 | 4.85 | 30800 | 22440 | 46.55 | 32.31 |
| 16 | 178 | 165 | 95 | 20 | 5.71 | 33200 | 20625 | 50.44 | 40.29 |
| 17 | 169 | 135 | 85 | 17 | 4.85 | 32300 | 16875 | 54.45 | 50.39 |
| 18 | 183 | 170 | 90 | 27 | 7.71 | 33700 | 28390 | 32.26 | 18.49 |
| 19 | 164 | 165 | 95 | 21.5 | 6.14 | 31800 | 25905 | 36.80 | 23.20 |
| 20 | 166 | 195 | 105 | 25.5 | 7.28 | 32000 | 26520 | 34.75 | 21.64 |
| 21 | 177 | 160 | 80 | 25 | 7.14 | 33100 | 26720 | 33.49 | 22.51 |
| 22 | 176 | 180 | 100 | 38.3 | 10.94 | 32000 | 30060 | 34.75 | 11.19 |
| 23 | 171 | 145 | 85 | 14 | 4 | 32500 | 14500 | 64.25 | 57.37 |

| | | | | | | | | | |
|---|---|---|---|---|---|---|---|---|---|
| 24 | 170 | 135 | 75 | 21 | 6 | 32400 | 18360 | 46.1 | 43.3 |
| 25 | 170 | 180 | 85 | 30 | 8.57 | 32400 | 30060 | 23.07 | 11.79 |
| 26 | 160 | 215 | 95 | 21 | 6 | 30400 | 32250 | 37.87 | 2.03 |
| 27 | 162 | 180 | 110 | 15 | 4.28 | 31600 | 15840 | 57.19 | 52.87 |
| 28 | 154 | 185 | 90 | 16 | 4.57 | 30800 | 23125 | 52.49 | 30.24 |
| 29 | 170 | 140 | 85 | 12 | 3.42 | 31400 | 14000 | 71.49 | 58.20 |
| 30 | 160 | 170 | 90 | 12 | 3.42 | 31400 | 17000 | 65.41 | 49.25 |
| 31 | 171 | 235 | 95 | 23 | 6.57 | 31500 | 36895 | 31.17 | 9.94 |
| 32 | 161 | 155 | 95 | 15 | 4.28 | 30500 | 24335 | 56.28 | 26.20 |
| 33 | 167 | 135 | 85 | 17 | 4.85 | 32100 | 11205 | 55.83 | 65.09 |
| 34 | 154 | 130 | 70 | 22 | 6.28 | 30800 | 13000 | 34.67 | 60.78 |
| 35 | 168 | 155 | 95 | 21 | 6 | 32200 | 14570 | 32.74 | 57.10 |
| 36 | 177 | 150 | 90 | 17 | 4.85 | 33100 | 22500 | 54.77 | 34.45 |
| 37 | 165 | 145 | 90 | 12 | 3.42 | 31900 | 19720 | 66.24 | 41.63 |
| 38 | 166 | 110 | 85 | 16 | 5 | 32000 | 18370 | 51 | 46.72 |
| 39 | 172 | 160 | 90 | 12 | 3.42 | 31600 | 21760 | 65 | 31 |
| 40 | 153 | 197 | 95 | 14.5 | 4.14 | 30700 | 19500 | 56 | 36.5 |
| 41 | 189 | 170 | 80 | 15 | 4.28 | 33300 | 24990 | 60 | 25 |
| 42 | 153 | 135 | 70 | 10 | 2.85 | 29700 | 20250 | 68 | 32 |
| 43 | 174 | 165 | 85 | 19 | 6.24 | 32800 | 24750 | 48 | 24.5 |
| 44 | 170 | 115 | 75 | 11 | 3.14 | 31400 | 13225 | 68 | 57.88 |
| 45 | 162 | 116 | 70 | 10 | 2.85 | 30600 | 9280 | 70 | 69 |
| 46 | 151 | 150 | 90 | 9 | 2.6 | 29500 | 14100 | 70 | 52 |
| 47 | 167 | 190 | 100 | 13.3 | 3.8 | 31100 | 30880 | 60 | 7 |
| 48 | 165 | 143 | 70 | 6 | 1.71 | 30900 | 16874 | 80 | 45 |
| 49 | 156 | 215 | 90 | 16.7 | 4.77 | 31000 | 32250 | 50 | 0 |
| 50 | 163 | 160 | 85 | 17 | 4.85 | 31700 | 16000 | 52 | 50 |
| 51 | 160 | 180 | 95 | 16.7 | 4.77 | 31400 | 27000 | 50 | 14 |
| 52 | 157 | 155 | 90 | 10 | 2.85 | 30100 | 17825 | 70 | 41 |
| 53 | 158 | 165 | 75 | 16 | 4.57 | 31200 | 24750 | 53 | 20 |
| 54 | 165 | 200 | 115 | 11 | 3.14 | 30900 | 17000 | 67 | 45 |
| 55 | 174 | 190 | 95 | 20 | 5.71 | 31800 | 20900 | 43 | 34 |
| 56 | 151 | 180 | 85 | 13 | 3.71 | 29500 | 16920 | 58 | 42 |
| 57 | 156 | 195 | 95 | 13.5 | 3.85 | 31000 | 32565 | 60 | 0 |
| 58 | 147 | 145 | 90 | 10 | 2.85 | 29100 | 16675 | 67 | 43 |
| 59 | 164 | 160 | 90 | 21 | 6 | 30800 | 21760 | 37.6 | 30 |
| 60 | 162 | 160 | 90 | 22.5 | 6.42 | 30600 | 18400 | 32.53 | 39.86 |
| 61 | 154 | 175 | 90 | 10.5 | 3 | 30800 | 21000 | 70 | 36 |
| 62 | 159 | 220 | 110 | 9 | 2.57 | 31300 | 29920 | 74 | 4 |
| 63 | 152 | 185 | 95 | 13.3 | 3.8 | 29600 | 23125 | 58 | 22 |
| 64 | 158 | 135 | 85 | 11 | 3.14 | 31200 | 18360 | 68 | 41 |
| 65 | 167 | 165 | 90 | 14 | 4 | 32100 | 19625 | 61 | 39 |
| 66 | 171 | 160 | 85 | 16.7 | 4.77 | 31500 | 27200 | 52 | 14 |
| 67 | 162 | 165 | 85 | 17 | 4.85 | 30600 | 17655 | 50 | 42 |
| 68 | 142 | 180 | 95 | 24 | 6.8 | 29600 | 18000 | 24 | 39 |
| 69 | 168 | 145 | 90 | 14 | 4 | 31200 | 18125 | 59 | 42 |
| 70 | 169 | 190 | 95 | 17 | 4.85 | 32300 | 31920 | 53 | 0 |
| 71 | 174 | 135 | 70 | 19 | 5.4 | 31800 | 17500 | 50 | 48 |
| 72 | 163 | 160 | 90 | 13.3 | 3.8 | 31700 | 24000 | 60 | 24.29 |

The data set has been manually classified by human experts on the basis of paraclinical and clinical investigations into three principal groups: valvular heart disease (VHD) and fibrillation of aortic insufficiency (FAI) (1-38); dilatative cardiomiopathy (DCM), arterial hypertension (AH), and congestive cardiac insufficiency (CCI) (39-48); ischemic cardiopathy (IC), effort pectoralis angina (EPA), and myocardium infarct (MI) (49-72).

**TABLE 3**
**Final Fuzzy Partition of the Cardiac Patients, without Data Normalization**

| Fuzzy class | Cardiac patients |
|---|---|
| $A_{111}$ | 8 9 28 32 43 53 63 72 |
| $A_{1121}$ | 12 19 20 51 66 |
| $A_{1122}$ | 14 18 21 41 |
| $A_{121}$ | 1 22 25 47 62 |
| $A_{1221}$ | 7 31 |
| $A_{12221}$ | 26 |
| $A_{12222}$ | 49 57 70 |
| $A_{2111}$ | 2 13 15 36 39 42 59 61 |
| $A_{2112}$ | 16 37 55 65 |
| $A_{2121}$ | 3 5 11 17 24 30 38 64 69 71 |
| $A_{2122}$ | 6 40 48 52 54 56 60 67 68 |
| $A_{221}$ | 4 27 50 58 |
| $A_{2221}$ | 10 23 29 34 35 44 46 |
| $A_{2222}$ | 33 45 |

The medical specialists who realized this classification noted that many patients showed symptoms indicating more than one illness. This fact, together with the large number of descriptors used, represents good support for using a fuzzy clustering approach.

### Fuzzy Hierarchical Classification of Cardiac Patients

The successive partition of the cardiac patients produced by using the 19 descriptors mentioned above is presented in Table 3. To ensure a more uniform participation of the various descriptors, we used a normalization (auto-scaling) of descriptors. The classification based on the same 19 descriptors but normalized—a procedure that avoids certain descriptors, expressed by larger numerical values, to prevail—gives finally just two classes. Table 4 shows the partition of the cardiac patient in this case.

**TABLE 4**
**Final Fuzzy Partition of the Cardiac Patients, with 19 Normalized Characteristics**

| Fuzzy class | Cardiac patients |
|---|---|
| $A_1$ | 1 2 5 7 8 9 11 12 13 14 15 16 18 19 20 21 22 24 25 26 28 31 32 35 36 38 43 47 49 51 53 55 57 59 60 66 68 70 |
| $A_2$ | 3 4 6 10 17 23 27 29 30 33 34 37 39 40 41 42 44 45 46 48 50 52 54 56 58 61 62 63 64 65 67 69 71 72 |

Comparing the results in Table 3 with the classes obtained by paraclinical and clinical investigations, we have observed certain differences. However, using data normalization the algorithm seems to produce better results providing only two classes: the first one including the majority of the valvular heart disease patients and the second one the majority of the dilatative cardiomiopathy and ischemic cardiopathy patients. In this case, a good agreement with the original diagnostics was established, as it may be seen in Table 5.

**TABLE 5**
**Original Diagnostic and Memberships to the Classes of the Final Fuzzy Partition Produced with Data Normalization (the illnesses order has been established by clinical analysis)**

| Patient | Illness | NYHA | $A_1$ | $A_2$ |
|---|---|---|---|---|
| 1 | VHD | 1 | 0.68002 | 0.31998 |
| 2 | VHD | 2/3 | 0.50467 | 0.49533 |
| 3 | VHD | 2/3 | 0.42350 | 0.57650 |
| 4 | VHD | 3 | 0.43222 | 0.56778 |
| 5 | VHD | 3 | 0.50049 | 0.49951 |
| 6 | VHD | 3 | 0.41299 | 0.58701 |
| 7 | VHD | 1 | 0.63278 | 0.36722 |
| 8 | VHD, AH | 2 | 0.56933 | 0.43067 |
| 9 | VHD | 2 | 0.76578 | 0.23422 |
| 10 | VHD | 3 | 0.44981 | 0.55019 |
| 11 | VHD | 2/3 | 0.53107 | 0.46893 |
| 12 | VHD | 2 | 0.71795 | 0.28205 |
| 13 | VHD | 2/3 | 0.55432 | 0.44568 |
| 14 | VHD | 1 | 0.62916 | 0.37084 |
| 15 | VHD | 3 | 0.62206 | 0.37794 |
| 16 | VHD | 2 | 0.57640 | 0.42360 |
| 17 | VHD | 3 | 0.40503 | 0.59497 |
| 18 | VHD | 1 | 0.63305 | 0.36695 |
| 19 | VHD | 2 | 0.69466 | 0.30534 |
| 20 | VHD | 1 | 0.58656 | 0.41344 |
| 21 | VHD | 1/2 | 0.65185 | 0.34815 |
| 22 | VHD | 1/2 | 0.60018 | 0.39982 |
| 23 | VHD | 3 | 0.38422 | 0.61578 |
| 24 | VHD | 2 | 0.61156 | 0.38844 |
| 25 | VHD | 1 | 0.64990 | 0.35010 |
| 26 | VHD, FAI | 2 | 0.56060 | 0.43940 |
| 27 | VHD | 2 | 0.47991 | 0.52009 |
| 28 | VHD, FAI | 2/3 | 0.51652 | 0.48348 |
| 29 | VHD, FAI | 3 | 0.33772 | 0.66228 |
| 30 | VHD | 2/3 | 0.49998 | 0.50002 |
| 31 | VHD, AH | 2/3 | 0.61521 | 0.38479 |
| 32 | VHD, MI, FAI | 3 | 0.56514 | 0.43486 |
| 33 | VHD, FAI | 2/3 | 0.38908 | 0.61092 |
| 34 | VHD, MI, FAI | 2/3 | 0.45803 | 0.54197 |
| 35 | VHD, AH | 2 | 0.51714 | 0.48286 |
| 36 | VHD | 2/3 | 0.62298 | 0.37702 |
| 37 | VHD | 3 | 0.45240 | 0.54760 |
| 38 | VHD | 3 | 0.52360 | 0.47640 |
| 39 | DCM, AH, CCI | 3 | 0.33589 | 0.66411 |
| 40 | DCM, AH, CCI | 3 | 0.37492 | 0.62508 |
| 41 | DCM, CCI | 3 | 0.45608 | 0.54392 |
| 42 | DCM, CCI, FAI | 3 | 0.38489 | 0.61511 |
| 43 | DCM | 2 | 0.50106 | 0.49894 |
| 44 | DCM, CCI | 3 | 0.37297 | 0.62703 |
| 45 | DCM | 3 | 0.37795 | 0.62205 |
| 46 | DCM, CCI | 3 | 0.36426 | 0.63574 |
| 47 | DCM, AH, CCI | 3 | 0.51120 | 0.48880 |
| 48 | AH, DCM, FAI, CCI | 3 | 0.40455 | 0.59545 |
| 49 | IC, EPA, AH | 2 | 0.63120 | 0.36880 |
| 50 | IC, EPA | 2/3 | 0.49107 | 0.50893 |
| 51 | IC, EPA, AH | 2 | 0.65957 | 0.34043 |

| | | | | |
|---|---|---|---|---|
| 52 | IC, EPA, CCI | 3 | 0.31495 | 0.68505 |
| 53 | IC, MI | 3 | 0.55236 | 0.44764 |
| 54 | IC, MI, AH, EPA | 3 | 0.39859 | 0.60141 |
| 55 | IC, MI, EPA | 2 | 0.50264 | 0.49736 |
| 56 | IC, EPA, MI | 3 | 0.34103 | 0.65897 |
| 57 | IC, EPA | 3 | 0.59843 | 0.40157 |
| 58 | IC, FAI | 3 | 0.38734 | 0.61266 |
| 59 | IC, MI | 2 | 0.68343 | 0.31657 |
| 60 | IC, EPA | 2 | 0.51521 | 0.48479 |
| 61 | MI | 3 | 0.43440 | 0.56560 |
| 62 | IC, EPA, AH | 3 | 0.48213 | 0.51787 |
| 63 | IC, AH, EPA | 3 | 0.40022 | 0.59978 |
| 64 | IC, EPA, AH | 3 | 0.27272 | 0.72728 |
| 65 | IC, EPA | 3 | 0.40367 | 0.59633 |
| 66 | MI | 2 | 0.65184 | 0.34816 |
| 67 | IC, EPA | 2/3 | 0.26617 | 0.73383 |
| 68 | IC, MI, EPA | 2 | 0.53834 | 0.46166 |
| 69 | MI, EPA | 3 | 0.34337 | 0.65663 |
| 70 | IC, EPA | 2 | 0.68006 | 0.31994 |
| 71 | IC, MI, EPA | 2 | 0.41871 | 0.58129 |
| 72 | IC, EPA | 3 | 0.45662 | 0.54338 |

The table also presents the NYHA functional class as it was established by paraclinical and clinical investigations. Moreover, considering the relatively large number of variables, we attempt, in the next section, to reduce it by applying a fuzzy clustering algorithm.

### Fuzzy Hierarchical and Horizontal Characteristics Clustering

The large number of available variables is always an issue, because of the extra computation required, and because not all the variables describe equally well the data. Following the fuzzy clustering of patients, our aim is to use fuzzy clustering in selecting the most relevant variables. We will next classify the data by using only these most relevant variables, and compare the results with those of the original fuzzy classification.

In order to develop the classifications presented in this section we applied the FHiCC procedure to the initial descriptors.

The characteristics clustering with the 19 descriptors for the 72 cardiac patients without data normalization produced the final partition presented in Table 6. The first descriptor separated from the others is 5, followed by 6 and then by 4. The cluster containing the descriptors from 7 to 19 is not subjected to any more splitting (their membership degrees, MD, to this cluster are all near 1). In the next step the descriptor 2 follows and, finally, 3 and 1.

The characteristics clustering with the same descriptors but with normalization (see Table 3) illustrates the same aspect, i.e., the high similarity of the last 13 descriptors based on effort testing and a large dissimilarity among the first descriptors including ECHO data and age and weight, respectively. This conclusion is supported also by the horizontal characteristics clustering, with the number of classes preset to seven, i.e., the number of classes produced by the hierarchic clustering procedure. The results of the horizontal characteristics clustering procedure are shown in Table 7. It is interesting to remark that all the divisions in Table 4 are clear-cut, the membership degrees to the different classes are all 1 or 0. We have to stress that the same treatment, but with a predefined number of eight classes, gives absolutely the same results, i.e., seven classes are clear-cut and one remains vacant, the MDs of all the descriptors to this class being zero.

**TABLE 6**
**Membership Degrees of the 19 Descriptors to the Clusters of the Final Fuzzy Partition without and with Data Normalization, Respectively**

| Descriptor | Without normalization | | | | | | | With normalization | |
|---|---|---|---|---|---|---|---|---|---|
| | $A_{111}$ | $A_{11211}$ | $A_{112121}$ | $A_{112122}$ | $A_{1122}$ | $A_{12}$ | $A_2$ | $A_1$ | $A_2$ |
| 1 | 0.218 | 0.210 | **0.526** | 0.000 | 0.044 | 0.000 | 0.000 | **0.537** | 0.463 |
| 2 | 0.260 | **0.643** | 0.003 | 0.002 | 0.090 | 0.000 | 0.000 | 0.498 | **0.502** |
| 3 | 0.171 | 0.052 | 0.000 | **0.681** | 0.094 | 0.000 | 0.000 | **0.561** | 0.439 |
| 4 | **0.991** | 0.002 | 0.001 | 0.001 | 0.003 | 0.000 | 0.000 | **0.528** | 0.472 |
| 5 | 0.001 | 0.000 | 0.000 | 0.000 | 0.000 | 0.002 | **0.996** | **0.589** | 0.411 |
| 6 | 0.000 | 0.000 | 0.000 | 0.000 | 0.000 | **0.759** | 0.241 | 0.495 | **0.504** |
| 7 | 0.002 | 0.000 | 0.000 | 0.000 | **0.997** | 0.000 | 0.000 | 0.008 | **0.992** |
| 8 | 0.002 | 0.000 | 0.000 | 0.000 | **0.997** | 0.000 | 0.000 | 0.008 | **0.992** |
| 9 | 0.002 | 0.000 | 0.000 | 0.000 | **0.997** | 0.000 | 0.000 | 0.008 | **0.992** |
| 10 | 0.002 | 0.000 | 0.000 | 0.000 | **0.997** | 0.000 | 0.000 | 0.008 | **0.992** |
| 11 | 0.002 | 0.000 | 0.000 | 0.000 | **0.997** | 0.000 | 0.000 | 0.008 | **0.992** |
| 12 | 0.002 | 0.000 | 0.000 | 0.000 | **0.997** | 0.000 | 0.000 | 0.008 | **0.992** |
| 13 | 0.002 | 0.000 | 0.000 | 0.000 | **0.997** | 0.000 | 0.000 | 0.008 | **0.992** |
| 14 | 0.002 | 0.000 | 0.000 | 0.000 | **0.997** | 0.000 | 0.000 | 0.008 | **0.992** |
| 15 | 0.002 | 0.000 | 0.000 | 0.000 | **0.997** | 0.000 | 0.000 | 0.008 | **0.992** |
| 16 | 0.002 | 0.000 | 0.000 | 0.000 | **0.997** | 0.000 | 0.000 | 0.008 | **0.992** |
| 17 | 0.002 | 0.000 | 0.000 | 0.000 | **0.997** | 0.000 | 0.000 | 0.008 | **0.992** |
| 18 | 0.002 | 0.000 | 0.000 | 0.000 | **0.997** | 0.000 | 0.000 | 0.008 | **0.992** |
| 19 | 0.002 | 0.000 | 0.000 | 0.000 | **0.997** | 0.000 | 0.000 | 0.008 | **0.992** |

**TABLE 7**
**Membership Degrees of the 19 Descriptors to the Seven Classes Obtained by Horizontal Classification**

| Descriptor | Fuzzy class | | | | | | |
|---|---|---|---|---|---|---|---|
| | $A_1$ | $A_2$ | $A_3$ | $A_4$ | $A_5$ | $A_6$ | $A_7$ |
| 1 | 0.000 | **1.000** | 0.000 | 0.000 | 0.000 | 0.000 | 0.000 |
| 2 | **1.000** | 0.000 | 0.000 | 0.000 | 0.000 | 0.000 | 0.000 |
| 3 | 0.000 | 0.000 | **1.000** | 0.000 | 0.000 | 0.000 | 0.000 |
| 4 | 0.000 | 0.000 | 0.000 | **1.000** | 0.000 | 0.000 | 0.000 |
| 5 | 0.000 | 0.000 | 0.000 | 0.000 | **1.000** | 0.000 | 0.000 |
| 6 | 0.000 | 0.000 | 0.000 | 0.000 | 0.000 | **1.000** | 0.000 |
| 7 | 0.000 | 0.000 | 0.000 | 0.000 | 0.000 | 0.000 | **1.000** |
| 8 | 0.000 | 0.000 | 0.000 | 0.000 | 0.000 | 0.000 | **1.000** |
| 9 | 0.000 | 0.000 | 0.000 | 0.000 | 0.000 | 0.000 | **1.000** |
| 10 | 0.000 | 0.000 | 0.000 | 0.000 | 0.000 | 0.000 | **1.000** |
| 11 | 0.000 | 0.000 | 0.000 | 0.000 | 0.000 | 0.000 | **1.000** |
| 12 | 0.000 | 0.000 | 0.000 | 0.000 | 0.000 | 0.000 | **1.000** |
| 13 | 0.000 | 0.000 | 0.000 | 0.000 | 0.000 | 0.000 | **1.000** |
| 14 | 0.000 | 0.000 | 0.000 | 0.000 | 0.000 | 0.000 | **1.000** |
| 15 | 0.000 | 0.000 | 0.000 | 0.000 | 0.000 | 0.000 | **1.000** |
| 16 | 0.000 | 0.000 | 0.000 | 0.000 | 0.000 | 0.000 | **1.000** |
| 17 | 0.000 | 0.000 | 0.000 | 0.000 | 0.000 | 0.000 | **1.000** |
| 18 | 0.000 | 0.000 | 0.000 | 0.000 | 0.000 | 0.000 | **1.000** |
| 19 | 0.000 | 0.000 | 0.000 | 0.000 | 0.000 | 0.000 | **1.000** |

We may conclude that the most significant descriptors, as shown by fuzzy clustering, are, in the order of their importance, 5 (ECHO data for right ventricle), 6 (ECHO data for left atrium), 4 (ECHO data for left ventricle), 2 (weight), 3 (ECHO data for left ventricle), 1 (age). This is because these descriptors have the highest discriminative power among the data set.

On the other side, the descriptors from 7 to 19 had the same membership degrees to all the produced classes, and this may indicate that they are representations of the same unique property. As such, we have added one of this descriptors to the set of six most relevant descriptors.

### Fuzzy Hierarchical Clustering of Cardiac Patients Considering Only Seven Characteristics

Taking into account the results obtained above referring to the characteristics clustering, it appears more illuminating and intuitive to use for the classification of cardiac patients only the first seven descriptors, namely age (1), weight (2), and ECHO data (3-7). In order to validate our method, we will use the same fuzzy clustering procedures, but on the data set described only by these seven descriptors.

The results obtained in this case without and with normalization are presented in Table 8. By careful examination and comparison with the results in Table 9 concerning the membership degrees of the patients to the four final classes it is easy to observe a good agreement with the classification based on the paraclinical and clinical examinations (see Table 5).

This analysis confirms that the data set using only the seven selected descriptors conserve its discriminative power, and supports our decision to discard the less relevant descriptors.

**TABLE 8**
**Final Fuzzy Partition of the Cardiac Patients Produced using Seven Characteristics without and with Data Normalization**

| Fuzzy class | Cardiac patients |
|---|---|
| | **Without data normalization** |
| $A_{11}$ | 3 4 5 6 8 9 16 17 22 31 32 35 38 40 49 51 53 59 61 66 |
| $A_{12}$ | 1 2 7 10 11 12 14 15 18 19 21 23 24 25 27 28 30 33 34 36 37 0 57 65 68 70 |
| $A_{21}$ | 13 20 26 39 47 54 55 56 60 62 63 64 67 71 72 |
| $A_{22}$ | 29 41 42 43 44 45 46 48 52 58 |
| | **With data normalization** |
| $A_{11}$ | 3 4 5 6 8 9 16 17 22 27 31 32 38 40 49 51 53 59 61 66 69 72 |
| $A_{12}$ | 1 2 7 10 11 12 14 15 18 19 21 23 24 25 28 30 33 34 35 36 37 50 57 65 68 70 |
| $A_{21}$ | 13 20 26 43 47 54 55 56 60 62 63 64 67 71 |
| $A_{22}$ | 29 39 41 42 44 45 46 48 52 58 |

**TABLE 9**
**Membership Degrees of the Cardiac Patients to the Classes of the Final Fuzzy Hierarchical Partition, without and with Data Normalization**

| Cardiac patient | Without normalization | | | | With normalization | | | |
|---|---|---|---|---|---|---|---|---|
| | $A_{11}$ | $A_{12}$ | $A_{21}$ | $A_{22}$ | $A_{11}$ | $A_{12}$ | $A_{21}$ | $A_{22}$ |
| 1 | 0.181 | 0.689 | 0.093 | 0.036 | 0.244 | 0.605 | 0.113 | 0.037 |
| 2 | 0.273 | 0.615 | 0.086 | 0.025 | 0.272 | 0.616 | 0.090 | 0.022 |
| 3 | 0.441 | 0.260 | 0.248 | 0.051 | 0.424 | 0.288 | 0.244 | 0.043 |
| 4 | 0.625 | 0.174 | 0.163 | 0.038 | 0.575 | 0.233 | 0.158 | 0.034 |
| 5 | 0.470 | 0.284 | 0.182 | 0.064 | 0.433 | 0.328 | 0.178 | 0.061 |
| 6 | 0.452 | 0.201 | 0.273 | 0.074 | 0.418 | 0.248 | 0.262 | 0.071 |
| 7 | 0.107 | 0.826 | 0.050 | 0.018 | 0.193 | 0.708 | 0.074 | 0.024 |
| 8 | 0.425 | 0.228 | 0.271 | 0.077 | 0.402 | 0.268 | 0.256 | 0.074 |
| 9 | 0.475 | 0.358 | 0.128 | 0.040 | 0.431 | 0.345 | 0.178 | 0.045 |
| 10 | 0.375 | 0.393 | 0.176 | 0.054 | 0.354 | 0.449 | 0.152 | 0.044 |
| 11 | 0.229 | 0.612 | 0.110 | 0.049 | 0.269 | 0.555 | 0.127 | 0.049 |
| 12 | 0.150 | 0.773 | 0.057 | 0.020 | 0.226 | 0.665 | 0.081 | 0.026 |
| 13 | 0.167 | 0.126 | 0.513 | 0.194 | 0.145 | 0.109 | 0.575 | 0.170 |
| 14 | 0.332 | 0.414 | 0.183 | 0.071 | 0.321 | 0.442 | 0.172 | 0.066 |
| 15 | 0.279 | 0.599 | 0.089 | 0.333 | 0.327 | 0.512 | 0.121 | 0.040 |
| 16 | 0.342 | 0.246 | 0.312 | 0.010 | 0.356 | 0.307 | 0.256 | 0.080 |
| 17 | 0.507 | 0.313 | 0.149 | 0.031 | 0.476 | 0.356 | 0.141 | 0.026 |
| 18 | 0.242 | 0.543 | 0.141 | 0.075 | 0.265 | 0.503 | 0.156 | 0.075 |
| 19 | 0.262 | 0.582 | 0.107 | 0.049 | 0.283 | 0.549 | 0.120 | 0.047 |
| 20 | 0.159 | 0.124 | 0.418 | 0.298 | 0.189 | 0.149 | 0.442 | 0.220 |
| 21 | 0.221 | 0.608 | 0.115 | 0.056 | 0.251 | 0.554 | 0.135 | 0.060 |
| 22 | 0.511 | 0.123 | 0.316 | 0.049 | 0.515 | 0.158 | 0.285 | 0.042 |
| 23 | 0.318 | 0.430 | 0.182 | 0.070 | 0.310 | 0.380 | 0.227 | 0.083 |
| 24 | 0.240 | 0.704 | 0.043 | 0.013 | 0.239 | 0.687 | 0.057 | 0.017 |
| 25 | 0.225 | 0.613 | 0.117 | 0.044 | 0.260 | 0.562 | 0.134 | 0.044 |
| 26 | 0.099 | 0.050 | 0.675 | 0.175 | 0.103 | 0.056 | 0.645 | 0.196 |
| 27 | 0.386 | 0.423 | 0.137 | 0.054 | 0.326 | 0.317 | 0.239 | 0.118 |
| 28 | 0.270 | 0.505 | 0.157 | 0.067 | 0.298 | 0.432 | 0.200 | 0.069 |
| 29 | 0.092 | 0.068 | 0.098 | 0.742 | 0.109 | 0.088 | 0.073 | 0.729 |
| 30 | 0.347 | 0.558 | 0.071 | 0.025 | 0.355 | 0.538 | 0.082 | 0.025 |
| 31 | 0.436 | 0.104 | 0.414 | 0.046 | 0.488 | 0.133 | 0.341 | 0.037 |
| 32 | 0.478 | 0.270 | 0.191 | 0.060 | 0.416 | 0.260 | 0.253 | 0.070 |
| 33 | 0.370 | 0.405 | 0.175 | 0.050 | 0.352 | 0.381 | 0.210 | 0.056 |
| 34 | 0.276 | 0.392 | 0.228 | 0.103 | 0.281 | 0.337 | 0.276 | 0.106 |
| 35 | 0.392 | 0.390 | 0.173 | 0.045 | 0.384 | 0.414 | 0.165 | 0.037 |
| 36 | 0.252 | 0.589 | 0.109 | 0.051 | 0.273 | 0.542 | 0.129 | 0.055 |
| 37 | 0.420 | 0.550 | 0.024 | 0.007 | 0.364 | 0.598 | 0.030 | 0.007 |
| 38 | 0.640 | 0.150 | 0.173 | 0.036 | 0.600 | 0.202 | 0.165 | 0.032 |
| 39 | 0.063 | 0.042 | 0.450 | 0.445 | 0.101 | 0.077 | 0.261 | 0.561 |
| 40 | 0.323 | 0.297 | 0.297 | 0.081 | 0.314 | 0.267 | 0.349 | 0.069 |
| 41 | 0.165 | 0.149 | 0.196 | 0.489 | 0.172 | 0.158 | 0.202 | 0.468 |
| 42 | 0.121 | 0.100 | 0.221 | 0.559 | 0.128 | 0.107 | 0.202 | 0.563 |
| 43 | 0.141 | 0.106 | 0.318 | 0.434 | 0.196 | 0.156 | 0.367 | 0.281 |
| 44 | 0.123 | 0.096 | 0.130 | 0.651 | 0.134 | 0.108 | 0.178 | 0.581 |
| 45 | 0.130 | 0.110 | 0.163 | 0.597 | 0.137 | 0.116 | 0.216 | 0.531 |
| 46 | 0.143 | 0.092 | 0.327 | 0.438 | 0.146 | 0.104 | 0.262 | 0.487 |
| 47 | 0.299 | 0.139 | 0.427 | 0.134 | 0.208 | 0.152 | 0.346 | 0.293 |
| 48 | 0.158 | 0.120 | 0.219 | 0.503 | 0.173 | 0.144 | 0.198 | 0.484 |

| | | | | | | | | |
|---|---|---|---|---|---|---|---|---|
| 49 | 0.471 | 0.333 | 0.144 | 0.051 | 0.446 | 0.344 | 0.158 | 0.052 |
| 50 | 0.400 | 0.481 | 0.093 | 0.026 | 0.362 | 0.437 | 0.154 | 0.047 |
| 51 | 0.562 | 0.181 | 0.207 | 0.050 | 0.452 | 0.289 | 0.203 | 0.056 |
| 52 | 0.081 | 0.049 | 0.358 | 0.512 | 0.094 | 0.061 | 0.386 | 0.459 |
| 53 | 0.460 | 0.151 | 0.317 | 0.071 | 0.476 | 0.189 | 0.276 | 0.059 |
| 54 | 0.148 | 0.081 | 0.550 | 0.221 | 0.118 | 0.071 | 0.599 | 0.212 |
| 55 | 0.104 | 0.065 | 0.625 | 0.205 | 0.223 | 0.148 | 0.507 | 0.121 |
| 56 | 0.197 | 0.128 | 0.504 | 0.171 | 0.190 | 0.129 | 0.524 | 0.157 |
| 57 | 0.281 | 0.584 | 0.096 | 0.039 | 0.310 | 0.542 | 0.109 | 0.038 |
| 58 | 0.138 | 0.116 | 0.202 | 0.543 | 0.151 | 0.131 | 0.184 | 0.535 |
| 59 | 0.572 | 0.267 | 0.126 | 0.035 | 0.520 | 0.304 | 0.140 | 0.036 |
| 60 | 0.179 | 0.071 | 0.703 | 0.047 | 0.159 | 0.074 | 0.715 | 0.051 |
| 61 | 0.567 | 0.268 | 0.128 | 0.036 | 0.510 | 0.304 | 0.147 | 0.038 |
| 62 | 0.261 | 0.123 | 0.444 | 0.171 | 0.276 | 0.145 | 0.427 | 0.153 |
| 63 | 0.169 | 0.087 | 0.578 | 0.167 | 0.199 | 0.111 | 0.542 | 0.149 |
| 64 | 0.125 | 0.061 | 0.761 | 0.052 | 0.201 | 0.096 | 0.664 | 0.038 |
| 65 | 0.332 | 0.437 | 0.176 | 0.055 | 0.333 | 0.455 | 0.166 | 0.046 |
| 66 | 0.461 | 0.313 | 0.162 | 0.063 | 0.442 | 0.298 | 0.195 | 0.065 |
| 67 | 0.067 | 0.043 | 0.663 | 0.226 | 0.095 | 0.062 | 0.705 | 0.139 |
| 68 | 0.286 | 0.430 | 0.188 | 0.096 | 0.298 | 0.379 | 0.218 | 0.105 |
| 69 | 0.526 | 0.140 | 0.286 | 0.048 | 0.533 | 0.190 | 0.237 | 0.039 |
| 70 | 0.143 | 0.803 | 0.041 | 0.013 | 0.185 | 0.742 | 0.056 | 0.016 |
| 71 | 0.107 | 0.054 | 0.752 | 0.086 | 0.167 | 0.087 | 0.671 | 0.074 |
| 72 | 0.330 | 0.136 | 0.449 | 0.084 | 0.419 | 0.184 | 0.334 | 0.062 |

### Fuzzy Horizontal Cardiac Patients Clustering Considering Only Seven Characteristics

We continue our analysis by clustering the set of patients with the seven descriptors without and with data normalization. Because the human experts indicated a classification of the patients in three classes, we will work here with the same number of classes. The fuzzy horizontal clustering distributes the cardiac patients according to the data presented in Table 10. It is interesting to remark in this case a better agreement with the classification obtained by paraclinical and clinical examinations. The class of arterial hypertension patients, $A_3$, is much better separated than the other ones. Concerning the class of valvular heart disease patients, $A_1$, and the class of the ischemic cardiac patients, $A_3$, each of them contains patients from the other one. However, we have to observe that in each of these classes we find the majority of patients indicated by paraclinical and clinical investigations (see Table 5) and this is a good validation of our technique.

The membership degrees of the cardiac patients to the classes of the final fuzzy partitions obtained by horizontal fuzzy clustering for seven descriptors without and with data normalization, presented in Table 11 illustrate also the efficiency of the fuzzy clustering approach. These fuzzy membership degrees are in good support with the medical practice that cardiac patients may present signs of more than one illness, since a clear-cut of the three groups of cardiac patients is practically impossible.

### Fuzzy Hierarchical Cross-Clustering

In what follows our aim is to identify the descriptors responsible with the separation of each class of patients. We will achieve this by using our fuzzy hierarchical cross-clustering algorithm on the set of 72 cardiac patients characterized by the same seven descriptors.

**TABLE 10**
**Results of Horizontal Fuzzy Clustering of the Cardiac Patients without and with Data Normalization, Considering only Seven Characteristics**

| Fuzzy class | Cardiac patients |
|---|---|
| | **Without data normalization** |
| $A_1$ | 1 2 7 9 10 11 12 14 15 18 19 21 23 24 25 27 28 30 33 34 35 36 37 49 50 57 65 66 68 70 |
| $A_2$ | 3 4 5 6 8 13 16 17 22 26 31 32 38 40 47 51 53 54 56 59 60 61 62 63 64 69 71 72 |
| $A_3$ | 20 29 39 41 42 43 44 45 46 48 52 55 58 67 |
| | **With data normalization** |
| $A_1$ | 1 2 7 9 10 11 12 14 15 18 19 21 23 24 25 27 28 30 33 34 35 36 37 49 50 57 59 61 65 68 70 |
| $A_2$ | 3 4 5 6 8 13 16 17 20 22 26 31 38 40 51 53 54 55 56 60 62 63 64 66 67 69 71 72 |
| $A_3$ | 29 39 41 42 43 44 45 46 47 48 52 58 |

**TABLE 11**
**Membership Degrees of the Cardiac Patients to the Classes of the Fuzzy Partition Obtained by Horizontal Fuzzy Clustering using only Seven Characteristics**

| Cardiac patient | Without normalization | | | With normalization | | |
|---|---|---|---|---|---|---|
| | $A_1$ | $A_2$ | $A_3$ | $A_1$ | $A_2$ | $A_3$ |
| 1 | 0.799 | 0.155 | 0.045 | 0.729 | 0.219 | 0.052 |
| 2 | 0.725 | 0.227 | 0.048 | 0.708 | 0.253 | 0.039 |
| 3 | 0.317 | 0.591 | 0.091 | 0.326 | 0.604 | 0.070 |
| 4 | 0.293 | 0.647 | 0.060 | 0.390 | 0.550 | 0.060 |
| 5 | 0.340 | 0.499 | 0.101 | 0.453 | 0.454 | 0.093 |
| 6 | 0.287 | 0.601 | 0.112 | 0.364 | 0.522 | 0.113 |
| 7 | 0.895 | 0.086 | 0.019 | 0.807 | 0.161 | 0.032 |
| 8 | 0.322 | 0.555 | 0.123 | 0.389 | 0.493 | 0.117 |
| 9 | 0.515 | 0.409 | 0.076 | 0.467 | 0.456 | 0.077 |
| 10 | 0.484 | 0.421 | 0.095 | 0.544 | 0.383 | 0.073 |
| 11 | 0.738 | 0.197 | 0.065 | 0.692 | 0.240 | 0.068 |
| 12 | 0.862 | 0.114 | 0.025 | 0.783 | 0.180 | 0.036 |
| 13 | 0.244 | 0.409 | 0.347 | 0.244 | 0.454 | 0.302 |
| 14 | 0.515 | 0.371 | 0.114 | 0.546 | 0.356 | 0.098 |
| 15 | 0.750 | 0.200 | 0.050 | 0.679 | 0.260 | 0.060 |
| 16 | 0.322 | 0.522 | 0.156 | 0.386 | 0.496 | 0.118 |
| 17 | 0.389 | 0.546 | 0.065 | 0.418 | 0.532 | 0.050 |
| 18 | 0.660 | 0.240 | 0.100 | 0.622 | 0.276 | 0.102 |
| 19 | 0.721 | 0.213 | 0.067 | 0.693 | 0.241 | 0.065 |
| 20 | 0.204 | 0.339 | 0.457 | 0.250 | 0.429 | 0.322 |
| 21 | 0.726 | 0.202 | 0.072 | 0.674 | 0.245 | 0.080 |
| 22 | 0.105 | 0.852 | 0.043 | 0.164 | 0.789 | 0.046 |
| 23 | 0.544 | 0.342 | 0.114 | 0.486 | 0.392 | 0.122 |
| 24 | 0.835 | 0.141 | 0.024 | 0.812 | 0.162 | 0.025 |
| 25 | 0.716 | 0.217 | 0.066 | 0.662 | 0.273 | 0.065 |
| 26 | 0.138 | 0.463 | 0.398 | 0.164 | 0.470 | 0.366 |

| | | | | | | |
|---|---|---|---|---|---|---|
| 27 | 0.577 | 0.334 | 0.089 | 0.446 | 0.389 | 0.165 |
| 28 | 0.635 | 0.264 | 0.100 | 0.568 | 0.328 | 0.104 |
| 29 | 0.052 | 0.089 | 0.860 | 0.051 | 0.080 | 0.869 |
| 30 | 0.740 | 0.217 | 0.043 | 0.731 | 0.228 | 0.040 |
| 31 | 0.067 | 0.896 | 0.036 | 0.109 | 0.854 | 0.036 |
| 32 | 0.408 | 0.491 | 0.101 | 0.386 | 0.504 | 0.110 |
| 33 | 0.512 | 0.395 | 0.093 | 0.478 | 0.431 | 0.091 |
| 34 | 0.517 | 0.325 | 0.158 | 0.464 | 0.379 | 0.157 |
| 35 | 0.478 | 0.435 | 0.087 | 0.494 | 0.439 | 0.066 |
| 36 | 0.715 | 0.216 | 0.069 | 0.668 | 0.256 | 0.076 |
| 37 | 0.810 | 0.168 | 0.021 | 0.828 | 0.155 | 0.017 |
| 38 | 0.231 | 0.717 | 0.051 | 0.304 | 0.646 | 0.050 |
| 39 | 0.100 | 0.214 | 0.686 | 0.111 | 0.191 | 0.698 |
| 40 | 0.408 | 0.443 | 0.149 | 0.376 | 0.499 | 0.124 |
| 41 | 0.171 | 0.219 | 0.610 | 0.178 | 0.226 | 0.596 |
| 42 | 0.114 | 0.171 | 0.715 | 0.117 | 0.171 | 0.712 |
| 43 | 0.153 | 0.265 | 0.582 | 0.237 | 0.381 | 0.382 |
| 44 | 0.089 | 0.141 | 0.771 | 0.109 | 0.169 | 0.722 |
| 45 | 0.110 | 0.159 | 0.730 | 0.131 | 0.192 | 0.676 |
| 46 | 0.141 | 0.284 | 0.575 | 0.140 | 0.238 | 0.622 |
| 47 | 0.216 | 0.591 | 0.193 | 0.243 | 0.374 | 0.383 |
| 48 | 0.147 | 0.233 | 0.619 | 0.163 | 0.224 | 0.612 |
| 49 | 0.494 | 0.418 | 0.087 | 0.514 | 0.401 | 0.084 |
| 50 | 0.622 | 0.326 | 0.054 | 0.564 | 0.360 | 0.076 |
| 51 | 0.265 | 0.663 | 0.072 | 0.409 | 0.501 | 0.090 |
| 52 | 0.088 | 0.207 | 0.705 | 0.118 | 0.252 | 0.630 |
| 53 | 0.196 | 0.712 | 0.091 | 0.269 | 0.649 | 0.084 |
| 54 | 0.175 | 0.462 | 0.362 | 0.186 | 0.459 | 0.354 |
| 55 | 0.162 | 0.408 | 0.429 | 0.240 | 0.555 | 0.206 |
| 56 | 0.234 | 0.457 | 0.309 | 0.251 | 0.473 | 0.276 |
| 57 | 0.733 | 0.210 | 0.057 | 0.706 | 0.238 | 0.055 |
| 58 | 0.132 | 0.185 | 0.683 | 0.138 | 0.185 | 0.677 |
| 59 | 0.448 | 0.484 | 0.067 | 0.492 | 0.443 | 0.064 |
| 60 | 0.132 | 0.706 | 0.162 | 0.166 | 0.668 | 0.165 |
| 61 | 0.446 | 0.486 | 0.067 | 0.487 | 0.445 | 0.067 |
| 62 | 0.195 | 0.564 | 0.240 | 0.232 | 0.555 | 0.212 |
| 63 | 0.173 | 0.505 | 0.321 | 0.213 | 0.527 | 0.260 |
| 64 | 0.148 | 0.608 | 0.244 | 0.155 | 0.722 | 0.123 |
| 65 | 0.536 | 0.367 | 0.097 | 0.556 | 0.368 | 0.075 |
| 66 | 0.464 | 0.434 | 0.102 | 0.445 | 0.454 | 0.101 |
| 67 | 0.143 | 0.336 | 0.520 | 0.192 | 0.476 | 0.332 |
| 68 | 0.558 | 0.303 | 0.139 | 0.513 | 0.338 | 0.149 |
| 69 | 0.151 | 0.794 | 0.055 | 0.257 | 0.684 | 0.059 |
| 70 | 0.890 | 0.092 | 0.017 | 0.846 | 0.132 | 0.022 |
| 71 | 0.148 | 0.564 | 0.288 | 0.168 | 0.665 | 0.166 |
| 72 | 0.175 | 0.695 | 0.130 | 0.230 | 0.682 | 0.088 |

The classification hierarchies produced in this way using both non-normalized and normalized data are presented in Table 12. The partitioning of the cardiac patients in classes 1 and 2 is practically the same in both cases. What is different is the partitioning of the descriptors in the two cases. The descriptors associated to the class 1 (without normalization), comprising the majority of valvular heart disease patients, are age (1), left ventricle (4), right ventricle (5), and left atrium (6). The patients in class 2 (without normalization), majority of arterial hypertension and ischemic cardiopathy patients, have as main descriptors the weight (2), left ventricle (3), and contractility (7).

**TABLE 12**
**Cross-Classification of the Cardiac Patients and Characteristics Produced with Seven Non-Normalized and Normalized Descriptors**

| Fuzzy class | Cardiac patients | Associated descriptors |
|---|---|---|
| | **Without data normalization** | |
| $A_1$ | 1 2 3 4 5 6 7 8 9 10 11 12 14 15 16 17 18 19 21 22 23 24 25 27 28 30 31 32 33 34 35 36 37 38 40 49 50 51 53 57 59 61 65 66 68 69 70 | 1 4 5 6 |
| $A_2$ | 13 20 26 29 39 41 42 43 44 45 46 47 48 52 54 55 56 58 60 62 63 64 67 71 72 | 2 3 7 |
| | **With data normalization** | |
| $A_1$ | 1 2 3 4 5 6 7 8 9 10 11 12 14 15 16 17 18 19 21 22 23 24 25 27 28 30 31 32 33 34 35 36 37 38 40 49 50 51 53 57 59 61 65 66 68 69 70 72 | 1 2 3 4 5 6 |
| $A_2$ | 13 20 26 29 39 41 42 43 44 45 46 47 48 52 54 55 56 58 60 62 63 64 67 71 | 7 |

In the case with data normalization, the main descriptor associated to the class 2 is only the contractility (7), the rest, namely age (1), weight (2), left ventricle (3) and (4), right ventricle (5) and left atrium (6) are classified with the class 1, which includes the majority of the valvular heart disease patients and half from ischemic cardiopathy patients. We remark again a good agreement with the medical observations presented in Table 5.

### CONCLUSIONS

Fuzzy classification algorithms applied to cardiac patients based on seven descriptors, namely ECHO data, and also age and weight, allow an objective interpretation of their similarities and dissimilarities. Moreover, the results obtained may be very useful in their reclassification. It is very interesting to study the classification of valvular heart disease and ischemic cardiopathy patients considering their membership degrees. Some of them belong practically with the same MD to the two classes, illustrating in this way the fuzziness of cardiac diseases. The new fuzzy approach, the fuzzy cross-classification algorithm, allows the qualitative and quantitative identification of the variables (descriptors) responsible for the observed similarities and dissimilarities between cardiac patients.

In addition, the fuzzy hierarchical characteristics clustering (FHiCC) and fuzzy horizontal characteristics clustering (FHoCC) procedures revealed a high similarity between the descriptors referring to the effort testing. This is one of the main conclusions and suggests their high redundant character concerning the diagnosis of cardiac diseases.
